# Association between *C1236T* Genetic Variant of *ABCB1* Gene and Molecular Response to Imatinib in Indonesian Chronic Myeloid Patients

**DOI:** 10.31557/APJCP.2019.20.11.3331

**Published:** 2019

**Authors:** Ikhwan Rinaldi, Riki Nova, Reni Widyastuti, Rizky Priambodo, Instiaty Instiaty, Melva Louisa

**Affiliations:** 1 *Department of Internal Medicine, Cipto Mangunkusumo Hospital,*; 2 *Clinical Pharmacology Fellowship Program, *; 4 *Department of Pharmacology and Therapeutics, Faculty of Medicine Universitas Indonesia, *; 3 *Human Genetic Research Center, Indonesian Medical Education, and Research Institute, Jakarta, Indonesia. *

**Keywords:** Imatinib, ABCB1, C1236T, chronic myeloid leukemia

## Abstract

**Objective:**

Imatinib is the first-line drug used for the treatment of chronic myeloid leukemia (CML) patients due to high molecular response and overall survival rate. However, some patients develop resistance to imatinib even after attaining a response. Mutation in *ABCB1 *efflux transporters is one of the known mechanisms of resistance to imatinib in chronic myeloid leukemia patients. This study was aimed to investigate the association of* ABCB1 C1236T* polymorphism in Indonesian chronic myeloid patients with molecular response to imatinib treatment.

**Methods:**

We analyzed 120 samples from chronic myeloid leukemia patients in the chronic phase, who had been on imatinib treatment for at least 12 months. We analyzed the *C1236T* variant of the *ABCB1* gene using PCR, followed by direct sequencing, and associate them with the achievement of major molecular response (MMR).

**Results:**

The major molecular response was achieved in 28% of patients. The frequencies of the SNPs were CC (40%), CT (46%), and TT (14%). Our result showed that there was a lack of association between polymorphism of *ABCB1* C1236T and imatinib response in Indonesian patients, with OR = 0.646 (95% CI: 0.283, 1.471; p>0.05).

**Conclusion:**

There was no association between *ABCB1 C1236T* variants with the major molecular response in Indonesian chronic myeloid leukemia patients receiving imatinib treatment.

## Introduction

Chronic myeloid leukemia (CML) is a disease of hematopoietic stem cells, characterized by the Philadelphia (Ph) chromosome and driven by its product, the *BCR-ABL1* tyrosine kinase (Hochhaus et al., 2017; Hochhaus et al., 2018). Imatinib is the first tyrosine kinase inhibitor available for the treatment of chronic myeloid leukemia. The drug acts by inhibiting the *BCR-ABL1* oncoprotein, which will result in the inhibition of phosphorylation of proteins involved in cell signal transduction (Cohen et al., 2005; Johnson et al., 2003). 

Upon treatment with imatinib, most patients restore normal hematopoiesis (Hochhaus et al., 2018). The long-term efficacy and safety imatinib in CML patients in the chronic phase have been established in IRIS Study (Hehlmann et al., 2017; Hochhaus et al., 2017; Hochhaus et al., 2018). After almost 11 years of follow-up, overall survival at ten years in imatinib-treated patients was 83.3% with no notable toxic effects (Hochhaus et al., 2017). Nonetheless, about 20 – 30 % of CML patients failed to obtain a complete hematological response and major molecular response (MMR) after treatment with imatinib (Milojkovic and Apperley, 2009). Several mechanisms of resistance to imatinib have been proposed, which include amplification of* BCR-ABL1*, mutations in the ABL-kinase domain, as well as mutation or overexpression in *ABCB1* efflux transporters (Milojkovic and Apperley, 2009; Zu et al., 2014). 

*ABCB1*, also known as P-glycoprotein or Multi-Drug Resistance 1 (MDR1), is a 170 – 190 kDa transmembrane protein that transports multiple endogenous compounds and xenobiotics. *ABCB1* involved in the absorption and elimination of many drugs. The action of *ABCB1* protein is to reduce intracellular drug accumulation via the efflux mechanism (Zu et al., 2014). *ABCB1* has been detected in CML patients and the circulating leucocytes in CML patients (Marzolini et al., 2004). *ABCB1* is highly polymorphic. Several studies had reported the most common variants in *ABCB1* gene, that are associated with imatinib response,* C1236T*, *C3435T *and* G2677T* in different populations (Angelini et al., 2013; Ben Hassine et al., 2017; Wang et al., 2015; Zu et al., 2014). However, the results had been inconclusive. Pooled data in a meta-analysis showed that in Asian CML patients, there is a significant association of imatinib resistance with *ABCB1 C1236T* variant, but not with *C3435T* and *G2677T* variant. Therefore, in the present study, we aimed to investigate whether the *C1236T* genetic variant is associated with major molecular response in Indonesian patients treated with imatinib.

## Materials and Methods

This was a nested case-control study, which included 120 stored whole blood samples from adult chronic myeloid leukemia patients in chronic phase treated with imatinib for at least 12 months. We compared those who failed to achieve major molecular response (case) and those who achieved MMR (control). Blood samples were collected from our previous research (Rinaldi et al., 2017). While Rinaldi et al., (2017) recruited 205 chronic myeloid leukemia patients, we only included samples from CML patients that have been treated with imatinib for at least 12 months. Our study was approved by the Ethics Committee Faculty of Medicine Universitas Indonesia/Ciptomangunkusumo Hospital, approval number 18-10-1194. All the samples were handled anonymously. Patients’ clinical data were collected from the medical records of the respective patients. Molecular response to imatinib is shown as the last available data of major molecular response (MMR) *(BCR-ABL/ABL* transcript ratio of less than 0.1%). 

*DNA Isolation*

DNA was isolated from whole blood samples using the Geneaid Blood DNA Isolation kit (Geneaid Biotech Ltd, Taiwan) according to the manufacturer’s instruction. Only DNA with high purity were confirmed using spectrophotometry were continued for PCR analysis. 

*C1236T ABCB1 genotyping*

Genotyping of the *C1236T* variant of *ABCB1* (*rs1128503*) was performed by PCR using MyTaq HS Red Mix (Bioline, UK), followed by direct sequencing using primers and methods described previously (Ben Hassine et al., 2017). Before sequencing, the PCR product length was on electrophoresis. PCR products were sequenced for forward directions using the same primer used for PCR amplification. BioEdit Sequence Alignment Editor version 7.2.1 was used for sequence analysis. 

*Statistical analysis*

Statistical analysis was performed using GraphPad Prism version 8.0. A chi-square test was done to test the significance of the genotype association with molecular response to imatinib treatment. The level of significance was set at p<0.05. Odds Ratio was calculated to determine the odds of having *ABCB1* *C1236T* variants in patients who failed in achieving MMR than those achieving MMR. 

## Results

From 120 adult patients with chronic myeloid leukemia (CML) in the chronic phase, the median duration of imatinib treatment was 19 months, and the median dose was 400 mg (ranged from 200 to 500 mg). Patients consist of 54 (45%) male and 66 (55%) female, with a mean age of 42.52 years old. The majority of patients (82.5%) were treated with hydroxyurea prior to treatment with imatinib. Out of the 120 CML patients, only 34 (28.3%) achieved MMR, while 86 (71.7%) patients failed to achieve MMR. 

**Figure 1:**
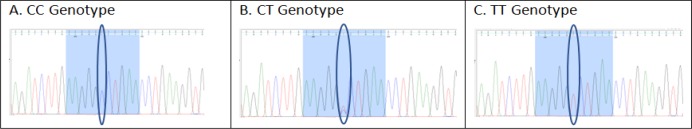
Domain Sequence for C1236T Variant of *ABCB1* in Patients with (A) Wild type CC Genotype (B) Mutant Heterozygote CT Genotype (C) Mutant Homozygote TT Genotype. Position of mutation is circled

Assessment of *C1236T ABCB1* genotype was analyzed using PCR followed by direct sequencing. The sample of results of domain sequence for the *C1236T* variant of *ABCB1* patients was given in Figure 1. 

The distribution of C1236T genetic variant of *ABCB1* consists of 40% wild type (CC); 45.8% have a heterozygous mutation (CT), and 14.2% have a homozygous mutation (TT).

We found no association between the *C1236T* variant of the *ABCB1* gene with major molecular response (Table 1).

## Discussion

Despite the radically improved outcome of CML patients treated with imatinib, about 20 – 30% of patients presented with resistance to this drug. Long term study with imatinib showed a 10-year survival of over 80% (Hehlmann et al., 2017; Hochhaus et al., 2017). However, our previous result in Indonesian patients showed a much lower response rate and survival (Reksodiputro et al., 2010). 

The role of *ABCB1* as efflux transporters of imatinib has been extensively studied, and thus far the results have been conflicting (Bedewy et al., 2019; Ben Hassine et al., 2017; Brambila-Tapia, 2013; Gromicho et al., 2013; Weiwei, 2012). 

Polymorphism of *ABCB1* has been studied widely as one of the mechanisms of imatinib resistance in CML patients. *ABCB1* or widely known as MDR1 or P-glycoprotein, is an ATP-driven efflux transporter that contributes to imatinib resistance by decreasing drug concentrations in the cells (Maia et al., 2018). Among many SNPs identified for *ABCB1*, 3 variants had been extensively studied for their association with imatinib treatment, *C1236T*,* G2677T* and *C3435T*, with inconclusive results (Angelini et al., 2013; Ben Hassine et al., 2017; Maia et al., 2018; Wang et al., 2015; Zu et al., 2014). A recent meta-analysis concluded that *MDR1 C1236T* polymorphism is the risk factor for the non-optimal clinical response in Asian CML patients (Zu et al., 2014). 

*ABCB1* variant *C1236T (rs118503)*, located in exon 12, is a synonymous type of polymorphism, that do not produce altered coding sequences (Brambila-Tapia, 2013). A study showed that this kind of mutation provides a modified function of *MDR1*. The change in the gene did not alter *MDR1 mRNA* expression. However, it changed the substrate specificity of the protein (Kimchi-Sarfaty et al., 2007). 

In contrast to other studies in CML patients treated with imatinib in different populations, our study showed low MMR achievement in CML patients after imatinib treatment (28.4%). The long duration of hydroxyurea treatment might explain the insufficient response to drug treatment in patients before imatinib treatment (Edesa and Abdel-malek, 2015; Rinaldi et al., 2017). We also found a high frequency of *ABCB1* C1236T polymorphism. In our study, we found 40% were CC, while the CT variant was 46%, and the TT variant was 14%. Our population showed a lower frequency of mutations compared to Thai, China or Korean populations (Ni et al., 2011; Pongstaporn et al., 2015; Seong et al., 2012; Yin et al., 2016). However, regarding the OR of the mutation in imatinib response, our result is quite similar to most Asian populations (Ni et al., 2011; Seong et al., 2012; Weiwei, 2012). We suspected that the higher frequency of homozygous mutation in other Asian populations, rather than heterozygous mutation type in our population, might be one the contributing factor. 

In the present study, we could not explain patients’ failure to achieve MMR with* C1236T* variant *ABCB1*. However, the presence of other drug transporters polymorphism has not been evaluated in our populations. Polymorphisms might not be things to be considered when we assess drug transporters. Studies had also shown that alterations in drug transporters’ expressions. Studies had shown that changes in gene expression did not only occurred with *MDR1*, but also *hOCT1*, *ABCG2*, *OCTN1*, and* OATP1A2* might explain the lack of response to imatinib treatment (Angelini et al., 2013; Chhikara et al., 2017; Rinaldi et al., 2017). 

Some studies demonstrated *ABCB1* haplotype, rather than individual SNPs, is a better predictor of imatinib response (Ali and Elsalakawy, 2014; Ben Hassine et al., 2017; Talaat et al., 2018; Vivona et al., 2014). While there were also reports that combinations of genetic variants in *ABCB1* with other drug transporters, mainly* OCT1* and *ABCG2* occurred in high frequency and lead to the reduced response of CML patients to imatinib (Galeotti et al., 2017; Yin et al., 2016). 

There are still possibilities of other mechanism of imatinib resistance in our population that are needed to be investigated further, such as the presence of *MDR1* haplotypes or the mutations in BCR-ABL kinase domain (Ben Hassine et al., 2017; Kaleemet al.,, 2015; Kimchi-Sarfaty et al., 2007; Milojkovic and Apperley, 2009).

In conclusion, our result suggested that the *C1236T *variant of *ABCB1* might not have an association with molecular response to imatinib in chronic myeloid leukemia patients. 

**Table 1:** Association between *C1236T* Variant of *ABCB1 *Gene with Major Molecular Response in CML Patients Treated with Imatinib

Genotype	Not achieving MMR	Achieving MMR	OR (95% CI)	p
CT + TT	49	37		
CC	37	49	0.646 (0.283-1.471)	0.282
TT	11	6		
CC + CT	75	28	0.684 (0.249-2.087)	0.563

## References

[B1] Ali MA, Elsalakawy WA (2014). ABCB1 haplotypes but not individual SNPs predict for optimal response/failure in Egyptian patients with chronic-phase chronic myeloid leukemia receiving imatinib mesylate. Med Oncol.

[B2] Angelini S, Soverini S, Ravegnini G (2013). Association between imatinib transporters and metabolizing enzymes genotype and response in newly diagnosed chronic myeloid leukemia patients receiving imatinib therapy. Haematologica.

[B3] Bedewy AML, Elmaghraby SM, Kandil NS (2019). Abcb1 and bmi1 mRNA expression in patients with chronic myeloid leukemia: Impact on imatinib efficacy. Blood Res.

[B4] Ben Hassine I, Gharbi H, Soltani I (2017). Molecular study of the ABCB1 gene and its correlation with imatinib response in chronic myeloid leukemia. Cancer Chemother Pharmacol.

[B5] Brambila-Tapia AJ (2013). Mdr1 (ABCB1) polymorphisms: Functional effects and clinical implications. Rev Invest Clin.

[B6] Chhikara S, Sazawal S, Seth T (2017). Molecular response to imatinib and its correlation with mRNA expression levels of imatinib influx transporter (oct1) in Indian chronic myeloid leukemia patients. Asian Pac J Cancer Prev.

[B7] Cohen MH, Johnson JR, Pazdur R (2005). U. S. Food and drug administration drug approval summary: Conversion of imatinib mesylate (sti571; Gleevec) tablets from accelerated approval to full approval. Clin Cancer Res.

[B8] Edesa WA, Abdel-malek RR (2015). Impact of imatinib interruption and duration of prior hydroxyurea on the treatment outcome in patients with chronic myeloid leukemia: Single institution experience. J Egypt Natl Canc Inst.

[B9] Galeotti L, Ceccherini F, Domingo D (2017). Association of the hoct1/ABCB1 genotype with efficacy and tolerability of imatinib in patients affected by chronic myeloid leukemia. Cancer Chemother Pharmacol.

[B10] Gromicho M, Magalhaes M, Torres F (2013). Instability of mRNA expression signatures of drug transporters in chronic myeloid leukemia patients resistant to imatinib. Oncol Rep.

[B11] Hehlmann R, Lauseker M, Saussele S (2017). Assessment of imatinib as first-line treatment of chronic myeloid leukemia: 10-year survival results of the randomized CML study iv and impact of non-CML determinants. Leukemia.

[B12] Hochhaus A, Larson RA, Guilhot F (2017). Long-term outcomes of imatinib treatment for chronic myeloid leukemia. N Engl J Med.

[B13] Hochhaus A, Saussele S, Rosti G (2018). Chronic myeloid leukemia: ESMP clinical practice guidelines for diagnosis, treatment, and follow-up. Ann Oncol.

[B14] Johnson JR, Bross P, Cohen M (2003). Approval summary: Imatinib mesylate capsules for the treatment of adult patients with newly diagnosed Philadelphia chromosome-positive chronic myelogenous leukemia in the chronic phase. Clin Cancer Res.

[B15] Kaleem B, Shahab S, Ahmed N, Shamsi TS (2015). Chronic myeloid leukemia--prognostic value of mutations. Asian Pac J Cancer Prev.

[B16] Kimchi-Sarfaty C, Oh JM, Kim IW (2007). A “silent” polymorphism in the mdr1 gene changes substrate specificity. Science.

[B17] Maia RC, Vasconcelos FC, Souza PS, Rumjanek VM (2018). Towards comprehension of the ABCB1/p-glycoprotein role in chronic myeloid leukemia. Molecules.

[B18] Marzolini C, Paus E, Buclin T, Kim RB (2004). Polymorphisms in human mdr1 (p-glycoprotein): Recent advances and clinical relevance. Clin Pharmacol Ther.

[B19] Milojkovic D, Apperley J (2009). Mechanisms of resistance to imatinib and second-generation tyrosine inhibitors in chronic myeloid leukemia. Clin Cancer Res.

[B20] Ni LN, Li JY, Miao KR (2011). Multidrug resistance gene (mdr1) polymorphisms correlate with imatinib response in chronic myeloid leukemia. Med Oncol.

[B21] Pongstaporn W, Pakakasama S, Chaksangchaichote P (2015). Mdr1 c3435t and c1236t polymorphisms: Association with high-risk childhood acute lymphoblastic leukemia. Asian Pac J Cancer Prev.

[B22] Reksodiputro AH, Syafei S, Prayogo N (2010). Clinical characteristics and hematologic responses to imatinib in patients with chronic phase myeloid leukemia (CML) at cipto mangunkusumo hospital. Acta Med Indones.

[B23] Rinaldi I, Reksodiputro A, Jusman S (2017). 323p association between duration of hydroxyurea administration before imatinib mesylate to the achievement of major molecular response (MMR) in the chronic phase of chronic myelocytic leukemia: A review on p-glycoprotein. Ann Oncol.

[B24] Seong S, Lim M, Sohn S (2012). Influence of enzyme and transporter polymorphisms on trough imatinib concentration and clinical response in chronic myeloid leukemia patients. Ann Oncol.

[B25] Talaat RM, Y K El-Kelliny M, El-Akhras BA (2018). Association of c3435t, c1236t, and c4125a polymorphisms of the MDR-1 gene in Egyptian children with acute lymphoblastic leukemia. Asian Pac J Cancer Prev.

[B26] Vivona D, Lima LT, Rodrigues AC (2014). ABCB1 haplotypes are associated with p-gp activity and affect a major molecular response in chronic myeloid leukemia patients treated with a standard dose of imatinib. Oncol Lett.

[B27] Wang JL, Liu HJ, Li F (2015). Multidrug resistance gene (mdr1) polymorphisms may not be directly associated with response to imatinib in chronic myeloid leukemia. Genet Mol Res.

[B29] Yin CX, Chen WW, Zhong QX (2016). Association between the concentration of imatinib in bone marrow mononuclear cells, mutation status of ABCB1, and therapeutic response in patients with chronic myelogenous leukemia. Exp Ther Med.

[B30] Zu B, Li Y, Wang X (2014). Mdr1 gene polymorphisms and imatinib response in chronic myeloid leukemia: A meta-analysis. Pharmacogenomics.

